# Gene Therapy of Thromboangiitis Obliterans with Growth Factor Plasmid (VEGF165) and Autologous Bone Marrow Cells

**DOI:** 10.3390/biomedicines12071506

**Published:** 2024-07-06

**Authors:** Piotr Barć, Paweł Lubieniecki, Maciej Antkiewicz, Diana Kupczyńska, Jan Barć, Katarzyna Frączkowska-Sioma, Tomasz Dawiskiba, Tadeusz Dorobisz, Wojciech Sekula, Błażej Czuwara, Małgorzata Małodobra-Mazur, Dagmara Baczyńska, Wojciech Witkiewicz, Jan Paweł Skóra, Dariusz Janczak

**Affiliations:** 1 Clinical Department of Vascular, General and Transplantation Surgery, Wroclaw Medical University, Borowska Street 213, 50-556 Wroclaw, Poland; barc.wroclaw@gmail.com (P.B.); maciej.antkiewicz@gmail.com (M.A.); k.fraczkowska.sioma@gmail.com (K.F.-S.); tomaszdawiskiba@gmail.com (T.D.); tdorobisz@gmail.com (T.D.); wsekula@tlen.pl (W.S.); jpskora@gmail.com (J.P.S.); dariusz.janczak@umw.edu.pl (D.J.); 2 Clinical Department of Diabetology and Internal Disease, Wroclaw Medical University, Borowska Street 213, 50-556 Wroclaw, Poland; 3 Ars Estetica-Clinic for Aesthetic Medicine and Laser Therapy, ul. Powstancow Ślaskich 56a/2, 53-333 Wroclaw, Poland; dr.diana.kupczynska@gmail.com; 4 Faculty of Medicine, Medical University of Lublin, Aleje Raclawickie 1, 20-059 Lublin, Poland; jan8876@gmail.com; 5 Department of Vascular Surgery, Provincial Hospital Center of the Jelenia Gora Valley, Oginskiego Street 6, 58-506 Jelenia Gora, Poland; blazej.czuwara@gmail.com; 6 Department of Forensic Medicine, Division of Molecular Techniques, Wroclaw Medical University, Borowska Street 213, 50-556 Wroclaw, Poland; malgorzata.malodobra-mazur@umw.edu.pl; 7 Department of Molecular and Cellular Biology, Wroclaw Medical University, Borowska 211A, 50-556 Wroclaw, Poland; dagmara.baczynska@umw.edu.pl; 8 Research and Development Center, Regional Specialized Hospital in Wroclaw, Kamienskiego 73a, 51-124 Wroclaw, Poland; sekretariat@wssk.wroc.pl

**Keywords:** thromboangiitis obliterans, gene therapy, VEGF, stem cells

## Abstract

Background: We performed gene therapy for critical limb ischemia in thromboangiitis obliterans (TAO) by the intramuscular administration of plasmids of the vascular endothelial growth factor gene (VEGF 165) with or without bone marrow-derived stem cells. Methods: The 21 patients were randomly assigned to three groups: A—with dual therapy, cells and plasmid; B—plasmid only; and C—control group, where patients received intramuscular injections of saline. Serum VEGF levels, the ankle–brachial index (ABI), transcutaneous oxygen pressure (TcPO2), and the rest pain measured by the visual analog scale (VAS) were determined sequentially before treatment, and then 1 and 3 months after treatment. Results: In the treatment groups, serum VEGF levels increased by 4 weeks and returned to baseline values after 3 months. ABI after 12 weeks increased by an average of 0.18 in group A, and 0.09 in group B and group C. TcPO2 increased by an average of 17.3 mmHg in group A, 14.1 mmHg in group B, and 10.7 mmHg in group C. The largest pain decrease was observed in group A and averaged 5.43 less pain intensity. Conclusions: Gene therapy using the VEGF plasmid along with or without bone marrow-derived mononuclear cells administered intramuscularly into an ischemic limb in TAO is a safe and effective therapy.

## 1. Introduction

Thromboangiitis obliterans (TAO) is an inflammatory disease leading to vascular obstruction that is not associated with atherosclerosis. The disease is strongly associated with smoking, which contributes to its onset and determines its course and prognosis [1]. TAO develops as an inflammation of the entire wall of smaller- and medium-sized vessels, both arteries and their accompanying veins, especially in the lower extremities [2,3]. The clinical consequence of TAO is limb ischemia, expressed by symptoms ranging from intermittent claudication to tissue necrosis and the loss of tissue integrity in the distal ischemic parts of the limbs. The development of critical limb ischemia awaits most patients, and the need for amputation affects more than a quarter of them [4,5,6]. Complete smoking cessation is a cornerstone of therapy. Local treatment, such as specialized dressings, is necessary to reduce pressure on the limb and prevent further injury. On the other hand, due to the anatomical location of vascular lesions, patients are rarely candidates for both endovascular treatment and open revascularization [7,8].

The failure of revascularization often results in an irreversible and unfavorable disease course [2,3,5,6,8]. Advances in molecular biology make it possible to develop new treatment strategies for TAO [9,10,11,12,13,14]. Taking advantage of the angiogenic effect of gene therapy with stem cells and growth factor gene plasmids (such as vascular endothelial growth factor—VEGF), new therapeutic options are being developed [15,16,17,18]. The expression of the angiogenic factor VEGF is induced under ischemic conditions and increases VEGF receptor-2-mediated endothelial progenitor cell mobilization [19,20]. Hewing et al. observed that there was less endothelial cell sprouting in the serum of TAO patients compared with controls, and, thus, less likelihood of neovascularization [21]. Based on our own experience with gene therapy for critical limb ischemia and data from the literature, we applied it as a partially blinded pilot study in patients with TAO [22,23,24,25]. The aim of the study was to evaluate the safety and feasibility, as well as efficacy, of treating critical limb ischemia in TAO with gene therapy via the intramuscular administration of the VEGF165 gene plasmid with or without mononuclear cells obtained from autologous bone marrow. The results are presented below.

## 2. Materials and Methods

### 2.1. Study Group

Nineteen men and two women were included in the study; the average age of the patients was 44 years (ranging from 31 to 59 years). Inclusion criteria were as follows:

1. TAO patients;
2. Critical ischemia of the lower limb (grades III and IV according to Fontaine’s classification) with rest pain and/or ulceration/arthrosis that has been healing for a minimum of 12 weeks;
3. No response to standard conservative treatment for at least 4 weeks;
4. Ineligibility for open or endovascular revascularization surgery.

Exclusion criteria are as follows:

1. Need for urgent amputation;
2. A history of cancer;
3. A history of severe, unstable retinopathy.

Objective documentation of ischemia was required. All patients had an ankle–brachial index (ABI) measurement performed at least twice before the study. In eligible patients, the ABI in the affected limb did not exceed 0.6 in two consecutive examinations performed up to 1 week apart. In addition, the patients described above were followed for 4 weeks on conventional pharmacotherapy to confirm that their clinical symptoms and objective ischemic parameters had not improved.

The study included 21 patients who were randomly assigned to three groups:

- A—patients received both the mononuclear cells and the VEGF165 plasmid;
- B—patients received VEGF plasmid in combination with saline;
- C—control group: patients received intramuscular injections of saline.

The study was partially blinded. Both the patient and the physician administering the intramuscular injections did not know which kind of medication was being administered. The blinding of the study did not apply to patients in group A, from whom bone marrow had previously been collected with shallow anesthesia under anesthetic control.

### 2.2. Study Protocol

The study was conducted between 2005 and 2020 and its protocol was approved by the Ethics Committee of the Medical University of Wroclaw (KB-926/2003).

All patients included in our study:

(1) met the inclusion criteria described above;
(2) did not have the exclusion criteria described above;
(3) were fully informed about the study and gave written informed consent.

The clinical study including blood pressure measurements, body temperature, and heart rate, as well as laboratory tests such as hematological, renal, hepatic, inflammatory, and metabolic parameters (morphology, C-reactive protein (CRP), creatinine, uric acid, urea, gamma-glutamyltranspeptidase (GGTP), alkaline phosphatase, alanine aminotransferase (ALAT), and aspartate aminotransferase (AspAT)) were determined before intervention, on day 1 or day 2, and after 1 week, 4 weeks, and 12 weeks. In view of the statistically insignificant results, they were omitted from the present study.

### 2.3. Bone Marrow Procurement and Preparation

Bone marrow was collected using commercial kit Harvest BMA C2. We collected 240 mL of bone marrow by multiple injections of iliac crest. Preparation was conducted strictly according to guidelines. It lasted approx. 30 min. After preparation (filtration and centrifugation), we archived 40 mL of solution including mononuclear cells (with their loss of no more than 30%). Mean amount of mononuclear cells: 18.8 ± 3.41 × 10,000,000/mL in bone marrow and 4.51 ± 0.9 × 1,000,000/mL in our solution. Mean amount of CD34+: 0.8 ± 0.18 × 1,000,000/mL in bone marrow and 0.183 ± 0.06 × 1,000,000/mL in solution. Loss of CD34+ was about 25%.

### 2.4. Production of VEGF Plasmid (phVEGF165)

For the treatment, we used a eukaryotic expression vector encoding the VEGF165 gene, corresponding to the NM_003376.6 sequence in NCBI [12,13]. The correctness of the DNA sequence was confirmed by the Sanger sequencing method and restriction analysis. Preparation and purification of the plasmid from cultures of phVEGF165-transformed Escherichia coli were performed with the endotoxin-free column method (Qiagen Mega Kit, Qiagen Inc., Valencia, CA, USA). The purified plasmid was stored in vials and pooled for quality-control analysis. Aliquots of 2000 µg of phVEGF165 were diluted in sterile saline to the volume of 10 mL before application.

In group A, a volume of 10 mL of solution containing 2 µg of plasmid 165 of the VEGF gene was added to a concentrate of mononuclear cells and incubated for about 30 min before administration.

### 2.5. Administration of the Preparation

In group A patients, immediately after the bone marrow was collected and prepared on the filters and centrifuge of the BAMAC kit (which took a total time of about 45–60′), a preparation of VEGF165 plasmid (about 2–4 µg) was added to the mononuclear cell preparation. After a short incubation in Dextran 40,000 with manual shaking lasting about 20 min, it was administered in numerous (average 80) intramuscular injections into the lower leg muscles. The depth of injection was 1.5–4.5 cm. Injection sites were determined based on blood supply and literature [26,27]. In order to produce as many collateral vessels as possible, the injections were performed at the border of the ones most affected by ischemia angiosomes. In all groups, the injections were performed in the same manner. Approximately 40 mL of preparation was administered. The volume of the administered agent for patients in groups B and C was about 20 mL.

Our intention was to eliminate the possible loss of stem cells caused by extracorporeal storage of bone marrow cells and to avoid prolonged exposure of the plasmid to enzymatic degradation by nucleases from monocytes present in the mononuclear cell solution. Therefore, the injection time did not exceed 2 h after bone marrow collection (usually, injections were performed 20 min after plasmids were added).

### 2.6. VEGF Serum Levels

Blood was collected from an upper-limb superficial vein before plasmid administration, on days 7, 14, 21, 28. and 90. Plasma was frozen after centrifugation. VEGF165 concentration was tested by ELISA using the R&D Systems kit according to the manufacturer’s instructions.

### 2.7. ABI before Treatment

The resting ankle–brachial ratio was calculated as the ratio of the lower of the pressures on the posterior or anterior tibial artery to the higher of the systolic pressures on the brachial arteries determined in the week before study administration and at 1 and 3 months after that.

### 2.8. Transcutaneous Oxygen Pressure (TcPO2) before Treatment

The Medicap Precise 8001 device was used for these measurements. It was determined at the same intervals as the ABI.

### 2.9. Visual Analog Scale (VAS) before Treatment

Resting pain was determined by patients on a 10-degree VAS scale before the injection procedure and at 4 and 12 weeks afterwards.

### 2.10. Imaging Studies

Patients underwent color duplex ultrasonography (CDU) of the arteries of the ischemic limb at the same intervals as ABI and TcPO2. Siemens Acuson Antares and Arietta 850 devices were used in the study. Flow was determined in the arteries of the lower extremities, especially flow spectra in axial vessels and collaterals. Angiotomography and classical arteriography were performed in each patient prior to gene delivery, in order to disqualify from classical revascularization therapy.

### 2.11. Statistical Analysis

Statistica 13.3 (StatSoft, Krakow, Poland) was used for statistical analysis. The work presents the results classified as the so-called industry statistics, using both descriptive statistics (age, gender, and yes/no data) and mathematical statistics. Paired chi-square and Wilcoxon tests were used to compare continuous variables before and after therapy and to evaluate the differences between the clusters of measurements taken at individual time points. *p* < 0.05 was considered significant.

## 3. Results

Group A, i.e., patients who were administered both the bone marrow preparation and VEGF165 plasmid, consisted of seven patients, including men alone. Group B, who were given the VEGF165 plasmid in combination with saline, also included seven participants, including six men and one woman. Control group C, who were administered saline alone, had seven individuals, including six men and one woman. Intramuscular injections were well-tolerated by patients in all groups. One participant in group A and one participant in group B experienced a transient increase in pain in the limb (up to 24 h), and four patients (one patient in group A, two patients in group B, and one patient in group C) experienced a transient, slight swelling of the limb that resolved within 24 h. One patient in Group B reported a pruritic sensation at the injection site, which resolved after 4 days, while two patients in Group A developed a fever, which resolved the next day. No serious complications were reported. There was no increase in leukocytosis or elevation of inflammatory markers (CRP). No significant decrease in morphology was observed in Group A patients after bone marrow collection. No significant changes in laboratory parameters were observed throughout the study (12 weeks). None of the patients was hospitalized during the 90-day follow-up period for reasons other than observation. No deaths or serious medical incidents were reported.

All patients received a conventional topical treatment, including the removal of necrotic tissue, wound debridement, the application of negative pressure therapy, and the use of appropriate dressing materials. In addition, the maximum relief of ischemic tissues was carried out. If infections were present, pharmacotherapy was administered with the targeted antibiotics selected based on the results of antibiogram cultures. The observation period was 3 months.

During this time, four major amputations were performed in the study patients due to worsening ischemia of the limb with a large infected ulcer. The amputations were performed between the 9th and 12th weeks of the study and all were below-knee amputations. This unfavorable course affected two patients in group C and one each in groups A and B. The complete healing of the ulceration was achieved in four patients (three patients in group A and one patient in group B), and partial healing occurred in six patients (one patient in group A, four patients in group B, and one patient in group C). In the remaining seven patients, the ulceration remained unchanged (two patients in group A, one patient in group B, and four patients in group C). The individual patient results are included in Table 1.

### 3.1. Changes in Serum Levels of VEGF165 in Patients

Changes in the endothelial growth factor levels affected patients in groups A and B. From a mean concentration of 258 ± 81 pg/L in the pre-treatment measurements, it increased at week 4 to a mean value of 389 ± 96 pg/L (*p* > 0.005), to drop to a mean value of 246 ± 74 pg/L in the study after 12 weeks. However, VEGF165 levels and their changes varied significantly and did not correlate with wound healing or treatment progression.

### 3.2. ABI after 12 Weeks of Treatment

**Figure 1:**
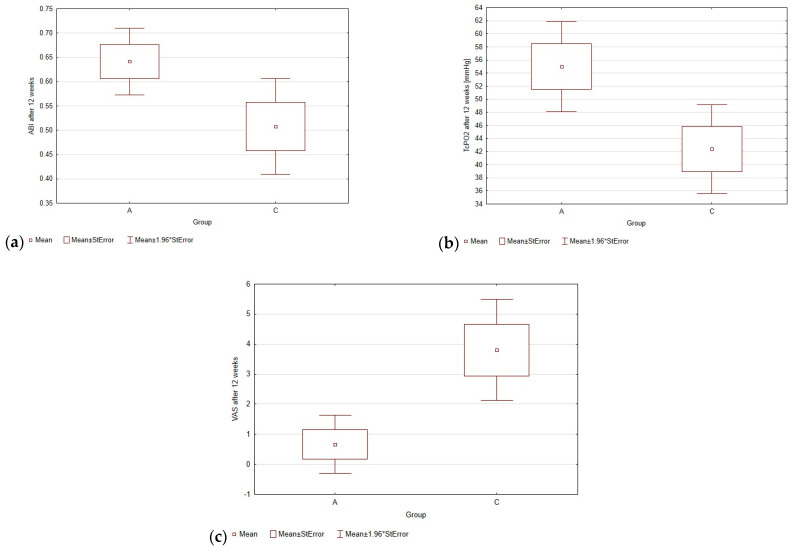
Comparison of ABI (**a**), TcPo2 (**b**), and VAS (**c**) values after 12 weeks in group A and control group C.

The increase in this parameter was observed after 12 weeks in group A and averaged 0.18 more with an average initial value of 0.46. In groups B and C, the increase was much lower, averaging 0.09 in each group with initial values of 0.44 and 0.42 in group B and group C, respectively. The statistically significant difference in results between groups A and C is shown in Figure 1. All the results of the analysis can be found in Table 2 and Table 3.

### 3.3. TcPO2 after 12 Weeks of Treatment

The largest increase in TcPO2 was observed in group A, and averaged 17.3 mmHg more after 12 weeks (the initial mean value was 37.7 mmHg). In Group B, the increase averaged 14.1 mmHg more (the initial mean value was 33.6 mmHg), while, in Group C, the increase was by far the lowest, almost two times lower than in Group A, and averaged 10.7 mmHg (the initial mean value was 31.7 mmHg).

### 3.4. VAS after 12 Weeks of Treatment

**Figure 2:**
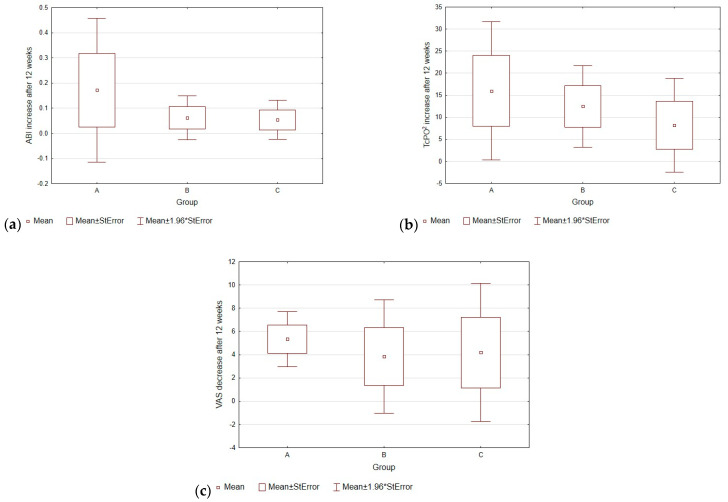
Average increase in ABI (**a**) and TcPo2 (**b**) values and decrease in pain intensity on the VAS scale (**c**) after 12 weeks in groups A, B, and C.

The subjective resting pain sensation decreased in all three groups. Patients in Group A had the greatest decrease, averaging 5.43 less pain intensity at a mean baseline of 6.1. Group B had an average of 4.0 less (mean baseline 6.3), while Group C had an average of 3.3 less (mean baseline 7.1). The trends in changes in mean ABI, TcPO2, and pain intensity on the VAS scale after 12 weeks are presented in Figure 2.

### 3.5. CDU

The baseline examination usually showed the closure of the popliteal artery and poorly developed collateral circulation. Follow-up examinations after 3 months showed the development of the collateral circulation vessels (the enlargement of existing ones and the appearance of new, small ones) and greater flow through subsequent sections of the main arterial trunks of the lower leg.

The follow-up classic angiography was abandoned due to its invasiveness and the fact that its results did not cause any change in therapy.

## 4. Discussion

It has been documented that the natural history of TAO has an inexorable descending course [4,5,6]. Therefore, amputation is often recommended as the solution of choice in patients with critical lower limb ischemia and progressive peripheral vascular damage due to TAO [7,8].

The good results obtained with VEGF gene therapy and the implantation of mononuclear cells obtained from the patient’s own bone marrow in the treatment of chronic lower extremity ischemia caused by atherosclerosis and diabetes, when surgical revascularization treatment is not possible, encouraged us to use this therapy in patients with TAO [16,17,18,23,26,27]. One reason was that surgical revascularization was not possible in patients with TAO. Another was that, when an obstruction of the middle artery led to ischemia, this caused the development of a collateral circulation, but was usually insufficient.

Proven “in vivo” on an animal model, the induction of angiogenesis by endothelial growth factor plasmids allows us to expect the same phenomenon in TAO patients. The administration of multipotent cells plays a similar role, providing material for the induction of angiogenesis. The accelerated development of collateral circulation can prevent amputation in patients. The gene therapies used in our study have just such an effect and, therefore, appear to be the best revascularization therapy available.

An analysis of the serum test results showed high initial levels of VEGF165 in all patients. It was statistically higher in all patients compared to healthy subjects. VEGF levels also showed a high variability from patient to patient. This situation appears to be due to the significantly increased production of VEGF protein by critical ischemic muscles in the affected limb [28]. During our 90-day follow-up period, the subjects experienced fluctuations in cytokine levels. It is noteworthy that, unlike previous reports using the VEGF gene alone, which showed that VEGF protein levels showed a transient peak in the systemic circulation 1 to 3 weeks after gene transfer, our study showed an increase in serum VEGF levels between 1 and 4 weeks after gene therapy [10,13]. The prolonged high VEGF levels in our study appear to be related to the increased cytokine production by transfected muscle cells at the injection sites, and we are hopeful that stem cell transfection from bone marrow cells will be successful [8,13,22]. However, changes in the VEGF levels were not statistically significant in our study. This seems to depend on the high and uncontrolled production of VEGF by ischemic muscles [28].

Thus far, trials of gene therapy with the VEGF plasmid or stem cells have been published, but there are no reports of the simultaneous use of these methods.

The patients tolerated the therapy very well. Complications were minor and were relatively rare. Aside from minor discomfort at the injection site in two cases, peripheral edema in four cases, pruritus in one patient, and fever in two cases, no other side effects were observed, and, in particular, there was no evidence of any systemic effects of VEGF on the development of retinopathy or new tumor growth in long-term follow-up. This treatment also does not hinder the typical treatment of patients with TAO.

It seems that, in the case of limb ischemia in TAO, when a medium-sized artery is amputated, it is necessary to obtain collateral circulation as soon as possible to salvage the ischemic tissues, both through collateral circulation from the thigh and adjacent, better vascularized vessels. A pro-angiogenic stimulus in the form of the administration of endothelial growth factor (VEGF) plasmids, as well as the delivery of mononuclear cells from the patient’s own bone marrow containing multipotent cells, enables the faster formation of capillaries, small vessels, and anastomoses, and, consequently, the formation of connections between the vasculature and the redistribution of the blood supply. In TAO, small vessels are not affected by the lesions. The sooner a collateral blood supply is established, the less likely necrosis and loss of the limb are. Gene therapy, therefore, makes it possible to avoid amputation. However, a condition for achieving success is the timing of its application. Since the activation of angiogenesis is a long-term process, in the case of advanced ischemic lesions, it is already too late. On the other hand, there is no contraindication to repeating the therapy later, if necessary.

Angiogenesis stimulated by gene therapy is confirmed subjectively by a reduction in pain intensity, and wound healing, and demonstrated by objective tests such as ABI, TcPO2, and imaging studies of the blood supply to the limb. In the group treated with VEGF and autologous bone marrow cells, the increase in ABI compared to the group receiving only VEGF and the control group was higher by almost three-fold. To the best of our knowledge, such an improvement in the ABI is difficult to achieve with drug treatment in patients with critical limb ischemia due to thrombophlebitis [29]. Similarly, a significant increase was obtained in the TcPO2 measurements, where the increase in the binary-treated group was two-fold higher compared to the control group. The above results are promising. We obtained an improvement sustained after 3 months of therapy, a reduction in the number of amputations and an increase in the percentage of healed ulcers compared to the control group.

In our study, we used only a plasmid of one gene, endothelial growth factor. We are currently studying bicistron plasmids containing the VEGF and hepatocyte growth factor (HGF) genes, as well as the angiopoietin 1 (ANG-1) plasmid. Synergistic effects on the induction of angiogenesis and maturation of newly formed vessels were studied in an animal model. Encouraging results were obtained, and another study on the use of different plasmids in patients with limb ischemia with TAO is underway. Initially, we also planned to select a group of patients treated with cells, but, due to technical and procedural difficulties related to the limited access for bone marrow harvesting and anesthetic protection, the inclusion of this group was abandoned. Two patients treated in this way outside the current study were not included. A cell-based study is planned for the future.

## 5. Conclusions

Gene therapy is a promising treatment method that stimulates angiogenesis. The application of the endothelial growth factor plasmid along with mononuclear cells obtained from autologous bone marrow is a safe and effective method of treating patients with ischemic, necrotic TAO lesions. However, late complications such as age-related macular degeneration (AMD) or malignancy cannot yet be determined. The patients are subject to further follow-up. The study requires continuation and needs to be expanded to include new plasmids with other growth factors.

## Figures and Tables

**Table 1:** Results of individuals patients. Patient groups: (A)—patients received both marrow preparation and VEGF165 plasmid, (B)—patients received VEGF plasmid in combination with saline, and (C)—control group: patients received saline by intramuscular injection. Ulceration: Amp.—amputation, 0—stationary, 1—healing, and 2—healed (after 12 weeks). x—no value in amputee patients.

Group	Sex	Age	ABI	ABI after 12 Weeks	TcPO2 [mmHg]	TcPO2 after 12 Weeks [mmHg]	VAS	VAS after 12 Weeks	Ulceration
A	M	35	0.35	0.6	44	60	5	0	1
A	M	50	0.62	0.65	44	67	6	0	2
A	M	44	0.46	0.55	34	43	5	1	0
A	M	33	0.52	0.65	34	51	7	0	2
A	M	40	0.4	x	30	x	7	x	Amp.
A	M	45	0.37	0.8	33	59	4	0	2
A	M	50	0.5	0.6	45	50	9	3	0
B	K	46	0.42	0.51	32	45	6	2	1
B	M	57	0.25	x	24	x	7	x	Amp.
B	M	46	0.45	0.49	40	59	6	1	2
B	M	49	0.7	0.7	43	48	5	6	1
B	M	56	0.46	0.57	35	50	6	1	1
B	M	41	0.35	0.38	26	36	7	3	0
B	M	38	0.45	0.55	35	48	7	1	1
C	M	41	0.58	0.62	29	34	6	7	0
C	M	31	0.32	0.35	25	40	8	3	0
C	M	59	0.37	x	25	x	5	x	Amp.
C	M	39	0.59	0.61	52	55	9	2	0
C	M	55	0.4	0.46	37	42	8	3	1
C	K	36	0.28	x	26	x	5	x	Amp.
C	M	36	0.38	0.5	28	41	9	4	0

**Table 2:** Analysis and comparison of results obtained in groups A and C after 12 weeks.

	Average Value in Group A	Average Value in Group C	SD in Group A	SD in Group C	*p*
ABI after 12 weeks	0.64167	0.50800	0.086120	0.112116	0.051801
TcPO2 after 12 weeks [mmHg]	55.00000	42.40000	8.602325	7.700649	0.032060
VAS after 12 weeks	0.66667	3.80000	1.211060	1.923538	0.009235

**Table 3:** Analysis and comparison of results obtained in groups B and C after 12 weeks.

	Average Value in Group B	Average Value in Group C	SD in Group B	SD in Group C	*p*
ABI after 12 weeks	0.53333	0.50800	0.105198	0.112116	0.708316
TcPO2 after 12 weeks [mmHg]	47.66667	42.40000	7.447595	7.700649	0.279663
VAS after 12 weeks	2.33333	3.80000	1.966384	1.923538	0.245016

## Data Availability

The data are available upon request.
